# Mining news media for understanding public health concerns

**DOI:** 10.1017/cts.2019.434

**Published:** 2019-10-23

**Authors:** Maryam Zolnoori, Ming Huang, Christi A. Patten, Joyce E. Balls-Berry, Somaieh Goudarzvand, Tabetha A. Brockman, Elham Sagheb, Lixia Yao

**Affiliations:** 1 Department of Health Sciences Research, Mayo Clinic, Rochester, MN, USA; 2 Center for Clinical and Translational Science, Community Engagement Program, Mayo Clinic, Rochester, MN, USA; 3 Department of Psychiatry and Psychology, Mayo Clinic, Rochester, MN, USA; 4 Mayo Clinic College of Medicine and Science, Rochester, MN, USA; 5 School of Computing and Engineering, University of Missouri-Kansas, Kansas City, MO, USA

**Keywords:** News, Reuters, Sentiment analysis, Topic modeling, Public health issue

## Abstract

**Introduction:**

News media play an important role in raising public awareness, framing public opinions, affecting policy formulation, and acknowledgment of public health issues. Traditional qualitative content analysis for news sentiments and focuses are time-consuming and may not efficiently convey sentiments nor the focuses of news media.

**Methods:**

We used descriptive statistics and state-of-art text mining to conduct sentiment analysis and topic modeling, to efficiently analyze over 3 million Reuters news articles during 2007–2017 for identifying their coverage, sentiments, and focuses for public health issues. Based on the top keywords from public health scientific journals, we identified 10 major public health issues (i.e., “air pollution,” “alcohol drinking,” “asthma,” “depression,” “diet,” “exercise,” “obesity,” “pregnancy,” “sexual behavior,” and “smoking”).

**Results:**

The news coverage for seven public health issues, “Smoking,” “Exercise,” “Alcohol drinking,” “Diet,” “Obesity,” “Depression,” and “Asthma” decreased over time. The news coverage for “Sexual behavior,” “Pregnancy,” and “Air pollution” fluctuated during 2007–2017. The sentiments of the news articles for three of the public health issues, “exercise,” “alcohol drinking,” and “diet” were predominately positive and associated such as “energy.” Sentiments for the remaining seven public health issues were mainly negative, linked to negative terms, e.g., diseases. The results of topic modeling reflected the media’s focus on public health issues.

**Conclusions:**

Text mining methods may address the limitations of traditional qualitative approaches. Using big data to understand public health needs is a novel approach that could help clinical and translational science awards programs focus on community-engaged research efforts to address community priorities.

## Introduction

Identifying sentiments and focuses of news media toward public health concerns is an emerging research topic of interest. News media play a substantial role in raising public awareness, framing public opinions, and affecting policy formulation and adoption of popular issues [1–3]. In the area of healthcare, news media use multiple channels to communicate evidence-based research findings to individuals and healthcare professionals and accelerate the translation of these research findings in healthcare to public health practice. For example, the news media have a drastic impact on changing the public’s perceptions, attitudes, and behaviors toward smoking, alcohol-impaired driving, and healthcare service utilization [4].

News media have a tendency to use language to influence the public’s opinions, behaviors, and perceptions related to specific health issues. For instance, antismoking articles emphasized the health risks of smoking with negative sentiment (e.g., fatal diseases such as lung cancer) and the benefits of quitting with positive sentiment (e.g., healthy life) using research findings and real patient cases [5]. The news media influence the understanding of public health concerns by selecting specific aspects of a topic and presenting the concerns as salient news articles [6].

Previous research assessing sentiments and the focus of news media toward public health were conducted using traditional qualitative content analysis. Glenn *et al*. analyzed the sentiments of national online news and the readers’ comments for weight loss surgery [7]. They found that the sentiments of the news articles were mostly positive and supportive, while the sentiments of readers’ comments were predominately negative and associated with some negative terms such as “piggy” and “fatty.” Patterson *et al*. analyzed the content of seven UK national newspapers to identify the style of presentation of news media for women’s and men’s drinking [8]. Their findings indicated a difference by participants’ gender. For instance, men’s drinking was mostly associated with the topics of “violence” and “disorderly,” while the women’s drinking was frequently linked to the topics “out of control,” “putting themselves in danger,” “harming their physical appearance,” and “burdening men.” Although providing useful insights into the sentiments and focuses of news media for a specific healthcare issue, qualitative content analytic approaches are costly, time-consuming, and resource intensive. There is the subjective nature of traditional qualitative inquiry due to human perceptions and interpretations. This subjectivity does not avail itself to efficiently or systematically detecting sentiments and the focus of news media. Another key factor related to the sample sizes used in the qualitative content analysis is usually limited to a few hundred of news articles, which could limit the generalizability of the findings.

To address these challenges, we used state-of-art text mining methods including sentiment analysis and topic modeling, together with statistical analysis, to efficiently analyze more than 3 million Reuters news articles to identify news coverage, sentiments, and emphases toward public health issues from 2007 to 2017. We identified 10 major public health issues (i.e., “air pollution,” “alcohol drinking,” “asthma,” “depression,” “diet,” “exercise,” “obesity,” “pregnancy,” “sexual behavior,” and “smoking”) based on the top keywords from public health scientific journals. Sentiment analysis refers to the use of computerized algorithms for systemically evaluating opinions (e.g., negative or positive sentiments) and their intensities of the words and sentences in a large collection of text documents [9]. Topic modeling employs computerized algorithms to automatically discover the hidden topics in a large body of text documents related to a specific subject.

The analysis of news media data with advanced text mining techniques allows the discovery of sentiments and focuses of news media for public health issues. These discoveries could shed light on the understanding of the most pressing health concerns and provide insight for public policy. Moreover, to our knowledge, no previous work used sentiment analysis and topic modeling for identifying sentiments and focuses of news media for public health issues.

## Methods

**Fig. 1.**
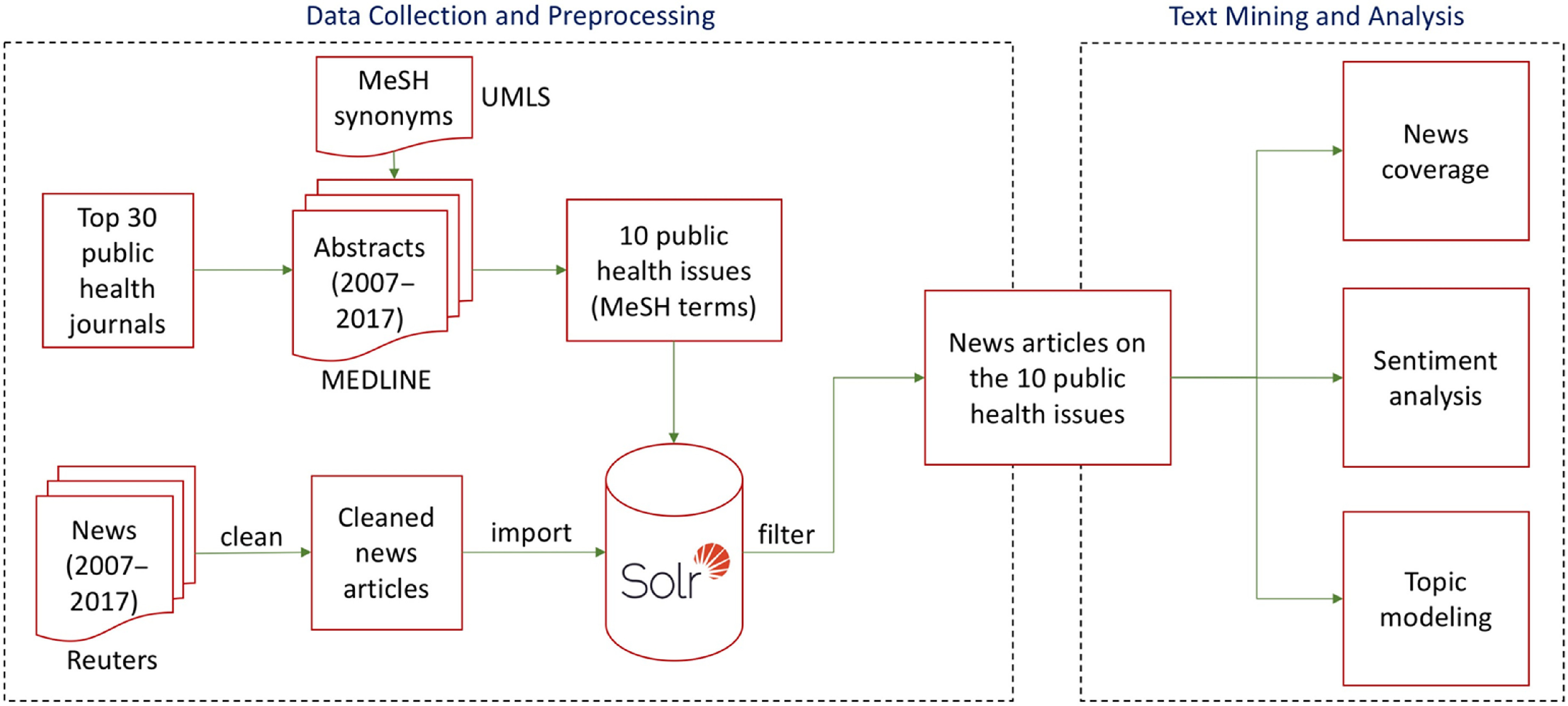
A schematic view of methods for mining Reuters news. MeSH, Medical Subject Heading; UMLS, Unified Medical Language System.

Fig. 1 shows a schematic view of the methods for mining Reuters news articles in this work. The methods consist of five main phases: (1) identifying the major public health issues from 30 top public health journals; (2) downloading, cleaning, and filtering news articles from Reuters news agency for the public health issues; (3) calculating the coverage of news articles over a decade linked to the public health issues and compare them with Google Trends searches; (4) analyzing sentiments of news articles related to the public health issues and their trends over time; and (5) identifying the focuses of the news articles associated with the public health issues. We briefly describe the five phases in the following subsections with detailed descriptions in Supplementary document 1. The Python scripts for mining news articles can be accessed via Github [10].

### Identifying Major Public Health Issues

Public health research studies generally investigate major public health issues to provide new knowledge and insights to increase wellness of the general population. These studies are mainly published in public health journals that are indexed in the MEDLINE database [11]. Abstracts of the published articles including the keywords present their main research focuses. Thus, we selected 30 of the top public health journals (See Supplementary document 2) and downloaded 61,387 abstracts of articles published between January 1, 2007 and December 31, 2017 to identify the major publish health issues.

We mapped the article keywords to Medical Subject Headings (MeSH) terms. MeSH is a controlled terminology developed by National Library of Medicine for indexing articles in the MEDLINE database [12]. We identified the synonyms of the MeSH terms using Unified Medical Language System (UMLS) Metathesaurus [13,14]. The UMLS Metathesaurus is a collection of controlled terminologies and provides mapping structures between different medical vocabularies via concept unique identifier. Sequentially, we developed a Python script with regression expression [10] to identify the MeSH terms (and their synonyms) in the abstracts and calculated the frequencies of the MeSH terms. The frequency of a MeSH synonym is added to the frequency of the MeSH term.

After removing the MeSH terms which were not related to public health or whose relative frequencies are less than 1%, we found 90 popular MeSH terms on public health (see Supplementary document 3). We selected 10 major public health issues (Table 1) out of the 90 MeSH terms based on the frequencies.

**Table 1.** Frequencies of MeSH terms related to public health

Public health issue (MeSH term)	Frequency
Smoking	4936
Pregnancy	3583
Obesity	1403
Air pollution	1009
Exercise	869
Diet	836
Sexual behavior	797
Alcohol drinking	788
Depression	755
Asthma	669

MeSH, Medical Subject Heading.

### Collecting, Cleaning, and Filtering News Articles

#### Collecting News Articles From Reuters News Agency

Reuters News Agency is a leading global information media agency and the world’s largest international text and television news provider [15]. We developed a web crawler in Python [10] to download news articles from an online archive of Reuters news agency [16]. We collected 3,763,737 articles between January 1, 2007 and December 31, 2017 to investigate major public health issues in the articles.

#### Cleaning News Articles

News articles contain noise and metadata that could affect the results of sentiment analysis and topic modeling toward public health issues. After reviewing a small sample of articles, we identified patterns that needed removing from the news articles. We removed repetitive special characters, such as “---”, from the articles. We also removed the tags, such as “(Reuters)” and editorial information (i.e., “reporting by” or “editing by”). We replaced the sentence delimiters “*” and “>” using a period and replaced the hyperlinks, such as “http://topnews.session.com” with the word “link.” In addition, readers’ comments were deleted in the articles since the focus of the analysis is the content of the story reported.

#### Filtering News Articles on Public Health Issues

To filter articles related to the 10 public health issues (Table 1), we imported the articles into Apache Solr [17] for information indexing and searching. Apache Solr is an open source search platform built on Apache Lucene library. Apache Lucene provides rich features to handle document such as full-text search and real-time indexing for various applications [18,19]. The Reuters news data were filtered in Apache Solr to retrieve articles that mentioned the 10 public health issues.

### News Coverage

Descriptive statistics were used to calculate the coverage of news media (i.e., numbers of news articles) for the 10 public health issues over time [20–23]. We compared the coverage trends of articles to Google Trends searches for the public health issues [20,21]. Google Trends analyzed the Internet search patterns of the individuals using Google search services over time [24]. The Internet search patterns reflect information-seeking behaviors of the individuals. Google Trends provides the majority of the Internet search services and makes the searching data publicly available.

### Sentiment Analysis

Sentiment is a view of or attitude (e.g., positive or negative opinion) toward a situation or event. Sentiment analysis denotes systematic evaluation of opinions and their intensities of the words and sentences in a large collection of text documents by using computerized algorithms [9]. Sentiment analysis is widely used in the area of healthcare [9,15,25,26], particularly for identifying the attitudes and opinions of patients’ posts in social media toward a specific healthcare issue. For example, Hopper and Uriyo used sentiment analysis to review patients’ feedback for a selected group of gynecologists in Virginia [27]. In another study, Clark *et al*. applied sentiment analysis to quantify sentiments of patients toward breast cancer treatment experience [28]. In our previous work, we also used sentiment analysis to identify the sentiments of news articles toward hundreds of diseases and medical conditions [15]. Computerized algorithms were developed to automatize the process of sentiment analysis, enabling researchers to evaluate sentiments of a large volume of text documents.

In this work, we used a python module, Valence Aware Dictionary and sEntiment Reasoner (VADER) [29], to quantify the sentiments of the articles toward the 10 identified public health issues. VADER was specifically tuned to identify the sentiments of a wide range of social media data [15,30]. VADER reports a normalized and weighted sentiment score for a given sentence, according to predefined score of each word and embedded rules. The reported sentiment score is between −1.0 (the most negative) and 1.0 (the most positive), with 0.0 indicating neutral.

To improve the accuracy of sentiment analysis for each public health issue, we measured the sentiments of sentences containing MeSH terms and their synonyms for the public health issue. We calculated the average of sentiment scores of all sentences related to the public health issue in articles as a sentiment score for the public health issue. We computed the average sentiment scores of all sentences linked to a public health issue in all the news articles as a sentiment score of news for the public health issue in each year. We classified an article as positive, neutral, or negative, according to the threshold values suggested by VADER [30]. More specifically, if a sentiment score of a news article is equal to or larger than 0.05, the sentiment of the news article is positive; if a sentiment score of a news article is less than 0.05 and larger than −0.05, the news article has a neutral sentiment; otherwise, the news article is negative.

### Topic Modeling

Due to the challenges (e.g., intensive human labor) of topic analysis with traditional manual qualitative methods, topic modeling methods were developed to automatically identify hidden topics in a massive collection of text documents [15,31,32]. The most frequently used topic modeling method is Latent Dirichlet Allocation (LDA) that was introduced by Blei *et al*. in 2003 [33]. LDA was extended and adopted in several domains for different purposes such as news themes on diseases [15], prognosis of human papillomavirus infection [34], and technology innovation in patents [31]. Although LDA is a powerful tool for discovering hidden topics in a large set of text documents, it is associated with some limitations. For example, LDA neglects the important word order in a text document. The text document is not treated as a sequence of words instead it is treated as a “bag” of words for topic modeling. LDA cannot automatically detect the number of topics in the text document and requires a predefined topic number as an input for topic modeling. Some posterior techniques such as perplexity and topic coherence [33,35] could help tune and find the appropriate number of topics in the text document, and they require extra computational time and involvement of domain experts to examine the generated topics for determining a good topic number. Given the number of topics, LDA infers topic distribution in each document (e.g., 0.5 * topic_1_ + 0.3 * topic_2_ + 0.2 * topic_3_ for the document_1_) and word distributions over a topic (e.g., 0.4 * word_1_ + 0.3 * word_2_ + 0.2 * word_3_ + 0.1 * word_4_ for the topic_1_). During the inference of word distribution over a topic, LDA treats a common word (i.e., a word occurs equally across all topics) and a characteristic word (i.e., a word occurs dominantly in a few topics) equally when they have the same probability given a topic.

In response to these limitations, we used an advanced topic modeling method, Topic Keyword Model (TKM) [36,37], to identify the hidden topic structure of articles related to the 10 public health issues. TKM considers the word order in a text document for topic modeling. From a human perspective, it seems that multiple consecutive words in a text document have large probabilities to associate with the same topic. Similarly, TKM links a word to a topic if it or its adjacent words have a high association score with the topic. Thus, the topic that a word links to is heavily influenced by its nearby words. This way, the order of words was involved in topic modeling with TKM, compared to LDA. TKM measures the dissimilarity between topics and only keeps the topics that are significantly different from each other during topic modeling. TKM could potentially determine the appropriate number of distinct topics in the text document. In addition, TKM differentiates a common word and a characteristic word for a topic and adjusted the association probability (score) of a word with a topic according to its commonness and distinctiveness among topics whiling inferring word distributions over a topic.

After word stemming and lemmatization that reduce words into their base forms [38], we used the TKM package provided by its author in GitHub [37] to learn the topics of the articles linked to each of the 10 public health issues. Because topic modeling algorithms are unsupervised learning methods and they do not require prior annotation as gold standard to train the models, the standard metrics (e.g., recall, precision, and F-measure) for supervised learning methods are not suitable for evaluating the results of topic modeling. Therefore, we asked domain experts to evaluate the results of topic modeling [39].

## Results

### Findings of News Coverage

**Fig. 2.**
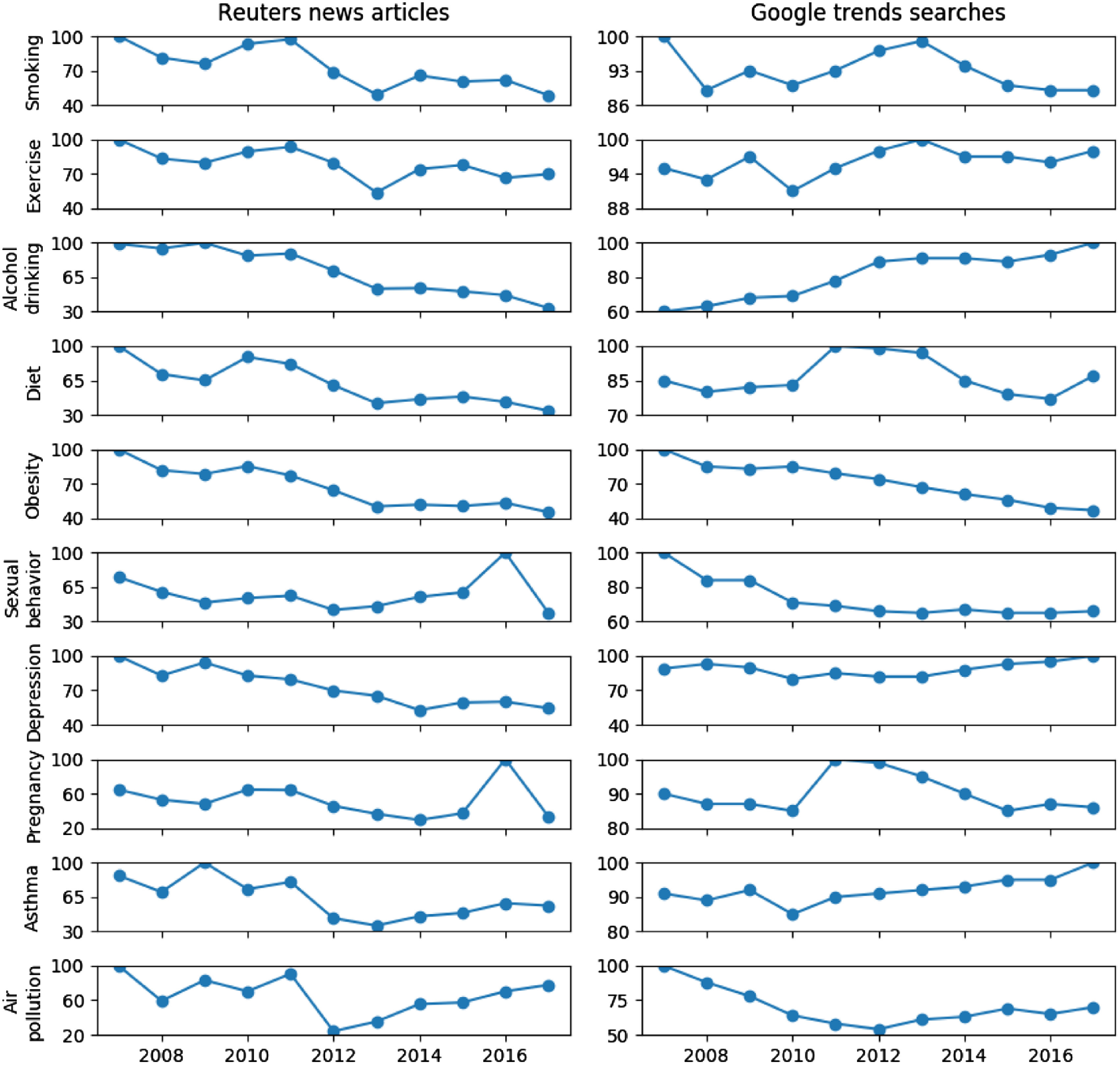
Normalized numbers of articles and Google Trends searches for the 10 public health issues over time. The numbers are normalized to the highest point on each subfigure. A value of 100 represents the peak popularity for the public health issue.

We calculated the coverage of articles associated with each of the 10 public health issues every year between 2007 and 2017. The results are compared with the numbers of Google Trends searches for the 10 public health issues as shown in Fig. 2. We rescaled these numbers relative to the highest number on each subfigure. Fig. 2 shows that the numbers of news articles for the seven public health issues “Smoking,” “Exercise,” “Alcohol drinking,” “Diet,” “Obesity,” “Depression,” and “Asthma” were constantly decreasing over years. The numbers of news articles for the remainder of the public health issues, “Sexual behavior,” “Pregnancy,” and “Air pollution” fluctuated in the study period. We found that the decreasing trends of Google searches for “Smoking” and “Obesity” are in line with the trends of relevant articles. In contrast, the number of Google searches for “Alcohol drinking” steadily increased over time, which had a negative correlation with the number of articles for “Alcohol drinking.”

### Findings of Sentiment Analysis

**Fig. 3.**
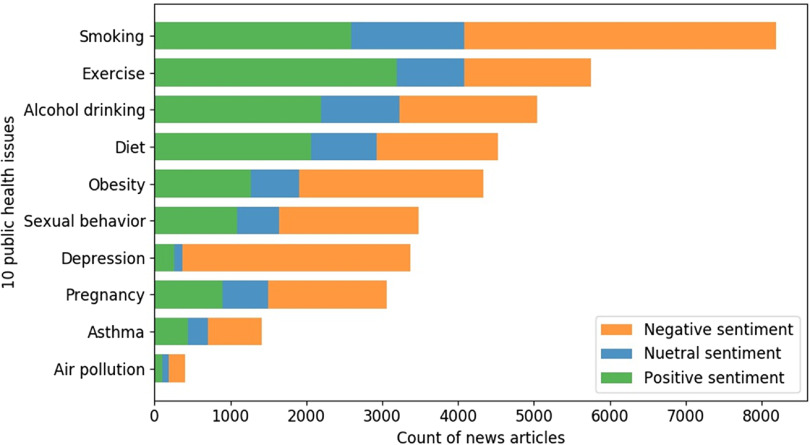
Counts of news articles with positive, neutral, and negative sentiments toward 10 public health issues.

Fig. 3 shows the frequencies of positive, neutral, and negative sentiments of the articles toward the 10 public health issues. We found that the sentiments of the news articles on the three of the public health issues, “exercise,” “alcohol drinking,” and “diet” were predominately positive (i.e., 55.6%, 43.4%, and 45.6%, respectively), implying that the articles associated these issues with positive terms, such as “happiness,” “energy,” or terms showing overall healthy life. For “alcohol drinking,” we found that there were more news articles with positive sentiment (43.4%) than news articles with negative sentiment (29.1%), which is surprising due to the public concern about alcohol misuse.

For the public health issues of “smoking,” “obesity,” “sexual behavior,” “depression,” “pregnancy,” “asthma,” and “air pollution,” Reuters published 50.0%–89.1% articles with negative sentiment and 8.9%–31.6% articles with positive sentiment. This occurred more frequently because the articles were linked the topics with negative terms, such as diseases, symptoms, low quality of life, “pressure,” “hopelessness,” and “worrying.” In addition, we found that among these public health issues, “smoking” is the mostly mentioned in the articles and “depression” had the largest coverage percentage (89.1%) of articles with negative sentiment.

**Fig. 4.**
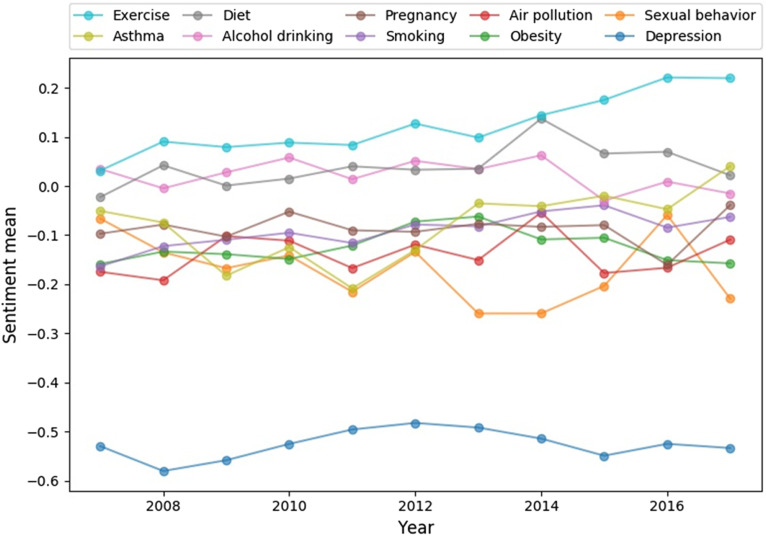
Sentiment scores of news media toward 10 public health issues over 11 years (2007–2017).

Fig. 4 shows the sentiment scores of the media toward the 10 public health issues over 11 years (2007–2017). During 2007–2017, “depression” had the lowest sentiment score that fluctuated between −0.6 and −0.4, compared with other public health issues. On the other hand, “exercise” showed the highest sentiment score after 2007 and its sentiment score steadily increased between 2007 and 2017. This finding could imply that exercise was increasingly linked to terms for reducing disease risk and improving life quality in the news articles.

### Findings of Topic Modeling

**Fig. 5.**
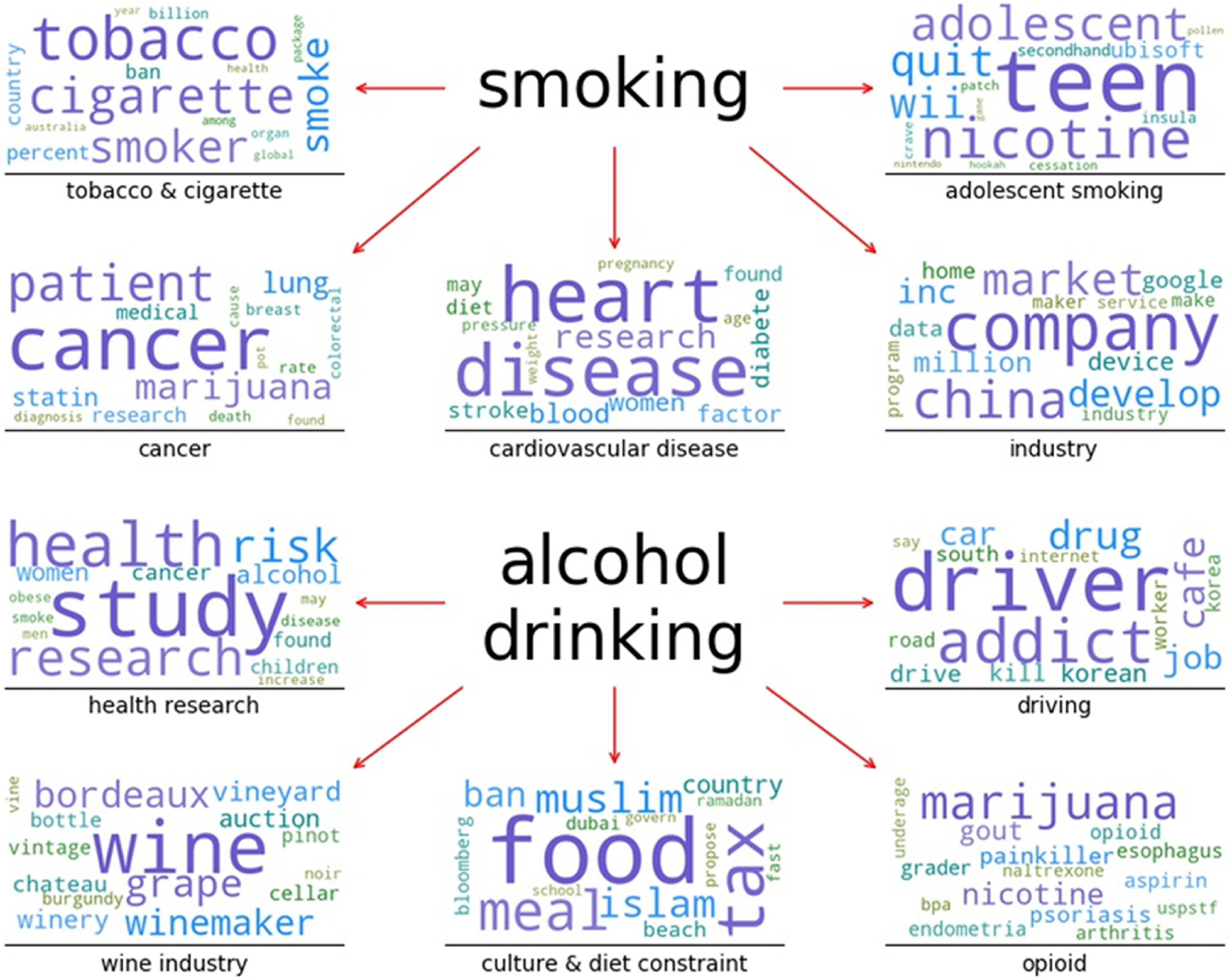
Word clouds of five meaningful topics identified in news articles related to the public health issues, “smoking” and “alcohol drinking.”

We used TKM to identify topics of articles associated with 10 public health issues. In this section, we presented the identified topics for two public health issues, “smoking” and “alcohol drinking” (Fig. 5). For the topics associated with the remaining eight public health issues, please see Supplementary document 4.

TKM identified 14 topics of the news articles related to “smoking.” We selected five most meaningful topics and showed them in Fig. 5 (see Supplementary document 4 for the rest nine topics). By interpretation of the identified topic keywords, we could find that the five meaningful topics of the news articles on “smoking” was mostly related to “tobacco and cigarette,” “industry,” “adolescent smoking,” “cancer,” and “cardiovascular disease.”

For articles on “alcohol drinking,” TKM discovered 16 topics and we selected and illustrated five most meaningful topics in Fig. 5. The remainder of the 11 topics is in Supplementary document 4. After interpretation of the identified topic keywords, we found that the five meaningful topics on “alcohol drinking” are “health research,” “driving,” “wine industry,” “culture and diet constraint,” and “opioid.”

## Discussion

We used descriptive statistics and state-of-art text mining techniques including sentiment analysis and topic modeling to identify the sentiments of over 3 million news articles toward public health issues and discover the hidden topic structures of these articles that discussed the issues. We selected 10 popular public health issues from 30 top public health journals indexed in the MEDLINE database. The 10 major public health issues are “air pollution,” “alcohol drinking,” “asthma,” “depression,” “diet,” “exercise,” “obesity,” “pregnancy,” “sexual behavior,” and “smoking.”

The coverage of articles associated with each of the seven public health issues, “Smoking,” “Exercise,” “Alcohol drinking,” “Diet,” “Obesity,” “Depression,” and “Asthma” had a declining trend over years. The decreasing trend of articles on “Smoking” is correlated with the trends of Google Trends searches and adult smoking prevalence in the US [40]. The reduced concern of the public media and individuals on “Smoking” were probably resulted from a series of smoking regulations and actions taken by the US government such as tobacco control initiatives since the 1960s [41]. The same trends of news articles and Google Trends searches were observed for the public health issue, “Obesity.”

The results of sentiment analysis showed that the sentiments of over 43% news articles toward “exercise,” “diet,” and “alcohol drinking” were positive. The dominantly positive sentiments “exercise” and “diet” could imply that the news articles mostly focused on the importance of healthy diet and regular exercise and their relationship with disease prevention and high quality of life. For example, articles such as “Study details how high fiber diets make for healthier lives” [42] and “Aerobic exercise eases depression, even in chronically ill” [43] linked the healthy diet and exercise to a healthy lifestyle and disease treatment. There is evidence indicating that “exercise” and/or “diet” (e.g., a Mediterranean diet) serve as a preventive or disease-modifying treatment of diseases such as dementia [44,45], Parkinson’s disease [46–48], and cardiovascular disease [49–51]. These studies possibly explain the constant increase of sentiment score of news media toward “exercise” over time. It seems to be surprising that the majority of the articles on “alcohol drinking” has positive sentiment, due to the public concern about excessive alcohol use. However, news articles such as “Drinking alcohol may keep leg arteries healthy” [52] and “Moderate drinkers have a better health, study finds” [53] showed how articles had a positive sentiment by highlighting the positive aspects of “alcohol drinking.” The attitude of articles toward “alcohol drinking” supported the findings by Mostofsky *et al*. [54]. According to their study, moderate alcohol drinking was associated with lower risk of cognitive decline and heart diseases. But moderate or higher alcohol intakes increase the risk of diseases such as breast cancer and bone fracture, particularly in women.

On the other hand, the sentiments of more than 50% articles were negative for “smoking,” “obesity,” “sexual behavior,” “depression,” “pregnancy,” “asthma,” and “air pollution.” The predominantly negative sentiments for these public health issues could be justified in two different ways: (1) the potential link of the public health issues to diseases, low work productivity, or poor quality of life. For example, articles such as “Air pollution a leading cause of cancer” [55], “Poor mental health harming productivity, says OECD” [56], and “Sexual harassment, abuse tied to real health effects” [57] linked “air pollution,” “mental disorder” (e.g., depression), and “inappropriate sexual behavior” to cancer, low productivity, and side effects on health, respectively. (2) The healthcare services and public health policies and programs are not effective enough to manage the issues. For example, articles such as “Kids with asthma often leave doctor’s office with unanswered questions” [58], “Obesity medical bill could reach $1.2 trillion a year by 2025” [59], and “Pot-smoking on the rise among U.S. pregnant women” [60] indicate the ineffectiveness of public health and healthcare interventions for managing “asthma,” “obesity,” and “pregnancy,” respectively. This is particularly the case for “depression” with the lowest sentiment score from the media, because it is currently one of the major public health concerns in our society and it is associated with several limitations in daily functioning and social participation [61].

We identified the main topics of the articles on the 10 public health issues using topic modeling. The highlighted five meaningful topics for two public health issues, “smoking” and “alcohol drinking.” The five major topics emphasized in the articles related to “smoking” were “tobacco and cigarette,” “industry,” “cancer,” “cardiovascular disease,” and “adolescent smoking.” The topics “cancer” and “cardiovascular disease” indicate that these fatal diseases were strongly associated with smoking in the media. It is well documented that smoking is linked to more than 12 cancers, particularly, lung cancer [62]. About 90% lung cancers are caused by tobacco smoking or secondhand smoke exposure [62]. Smoking is linked to cardiovascular health and cardiovascular disease with smoking attributing to about 140,000 premature deaths annually [63]. The topic “adolescent smoking” suggests that tobacco use in adolescence is another focus of the articles. In 2013, about 18% middle school students and 46% high school students were tobacco users [64]. Nearly 90% cigarette smokers first tried cigarette smoking by age 18, and the prevention of tobacco use in adolescence is critical to reducing tobacco epidemic in the US [41,65].

The five major topics uncovered in the articles on “alcohol drinking” are “health research,” “driving,” “wine industry,” “culture and diet constraint,” and “opioid.” The topic “health research” may show that health-related research on “alcohol drinking” was mainly discussed in the articles, for example, the news articles “Cutting back on alcohol can prevent cancers: experts” [66] and “Moderate drinking helps heart, but don’t binge” [67]. The topic “opioid” suggests the mixed use of opioid and alcohol and related risks. The opioids are effective painkiller but have the potential to be additive [68]. The combined use of opioid and alcohol increases the risk of overdose and injury [69]. The topic “driving” implies that the alcohol-impaired driving was another focus of many of the articles, possibly because driving under the influence of alcohol remains a public health problem [70].

The use of big data to understand public health issues is a novel way to provide clinical and translational science awards programs insight on community priorities that lend themselves to community-engaged research approaches [71]. Our other research indicates that meaningful community engagement offers the opportunity to promote bi-directional dialogue about health research with diverse communities [71–73]. The process used in this study offers a new way to identify topics for future dialogs with community-engaged stakeholders to set research priorities.

The discussion of this study cannot be considered fully without analyzing its limitations. The first limitation is related to the data source. We used articles published by Reuters between 2007 and 2017 for studying major public health issues because Reuters is a leading news media organization and the largest international text news provider. Although we are confident that the findings are not atypical for other media sources that cover public health issues, the results of coverage, sentiment analysis and topic modeling might not be generalized to other news media agencies (e.g., Associated Press) or other forms of media (e.g., television, radio or social media). In addition, the findings of news media may not reflect the concerns of the US population which is another indication that the findings lend themselves for starting a dialog about these topics as potential areas of focus for health-related research with the community.

The second limitation of the study is the synonyms generated using UMLS for each public health issue. Since UMLS is a compendium of standard medical terminologies, it might not include all synonyms that Reuters journalists use when writing about public health issues.

The third limitation relates to the sentiment analysis method (VADER) used for identifying sentiments of articles toward the public health issues. Although VADER has been evaluated using articles published by New York Times, it has not been tested or tuned on news articles from Reuters.

The fourth limitation is about the topic modeling method. We used TKM to identify topics in news articles for each public health issue. Although TKM addresses the limitations of LDA, topic modeling with TKM was performed in a completely unsupervised fashion. To evaluate the results of topic modeling, we relied on domain experts’ judgments that might create bias in interpretation of these topics.

## Conclusion

In this study, we identified 10 important public health issues after analyzing more than 60,000 abstracts of 30 top public health journals. We analyzed over 3 million Reuters articles during 2007–2017 identified their sentiments with linkages to the 10 public health issues, using state-of-art text mining methods including sentiment analysis and topic modeling.

Our results show that the coverage of news articles associated with each of the seven public health issues, “Smoking,” “Exercise,” “Alcohol drinking,” “Diet,” “Obesity,” “Depression,” and “Asthma” had a declining trend over years. The coverage of news articles for the rest three public health issues, “Sexual behavior,” “Pregnancy,” and “Air pollution” fluctuated over time. For sentiment analysis, the sentiments of the news articles for the three public health issues, “exercise,” “alcohol drinking,” and “diet,” were predominately positive. It suggests that the articles associated these issues with positive terms such as energy. For the remainder of the seven public health issues including “smoking,” “obesity,” “sexual behavior,” “depression,” “pregnancy,” “asthma,” and “air pollution,” most articles had negative sentiments. It may indicate that the articles mostly linked these issues to negative terms such as diseases or symptoms.

Our study showed that text mining methods may address the limitations associated with traditional qualitative approaches. Our analysis could provide valuable insights about the sentiments and topic structures of articles discussing public health issues. Our findings could offer valuable information for the healthcare professionals and policy makers.

## Supplementary material

For supplementary material accompanying this paper visit https://doi.org/10.1017/cts.2019.434. click here to view supplementary material
