# Supplementary material for: Mining news media for understanding public health concerns
Source: J Clin Transl Sci. 2019 Oct 23;5(1):e1. doi: 10.1017/cts.2019.434 (PMC8057471; doi:10.1017/cts.2019.434)
Supplement: Supplementary file 1 [file S2059866119004345sup.zip › S2059866119004345sup002.docx]

**Mining News Media for Understanding Public Health Concerns**

**Supplementary Document**

Maryam Zolnoori, PhD^1^^[[1]](#footnote-1)^, Ming Huang, PhD^1*^, Christi A. Patten, PhD^2,3^, Joyce E. Balls-Berry, PhD, MPE^1,4^, Somaieh Goudarzvand^5^, Tabetha A. Brockman, MA^2,3^, Elham Sagheb Hossein Pour, MS^1^, and Lixia Yao, PhD^1^

^1^Department of Health Sciences Research, Mayo Clinic, Rochester, MN, United States

^2^Center for Clinical and Translational Science, Community Engagement Program, Mayo Clinic, Rochester, MN, United States

^3^Department of Psychiatry and Psychology, Mayo Clinic, Rochester, MN, United States

^4^Mayo Clinic College of Medicine and Science, Rochester, MN, United States

^5^School of Computing and Engineering, University of Missouri-Kansas, Kansas City, MO, United States

**Corresponding Author:**

Lixia Yao, PhD

Department of Health Sciences Research

Mayo Clinic

200 First Street SW

Rochester, MN, 55905

United States

Phone: 1 507-293-7953

Fax: 1 507-284-1516

Email: [Yao.Lixia@mayo.edu](mailto:Yao.Lixia@mayo.edu)

All authors have no conflict of interest to declare.

**Technical Steps to Probe Public Health Issues with Reuters News Articles**

We used descriptive statistics and cutting-edge text mining methods including sentiment analysis and topic modeling to efficiently analyze more than 3 million Reuters news articles between 2007 and 2017 for identifying their coverage, sentiments, and focuses for major public health issues. The main steps for mining Reuters news articles mainly include:

1. Identifying major public health issues from articles published in 30 top public health journals
2. Downloading, cleaning, and filtering news articles from Reuters news agency
3. Calculating the coverage of news articles linked to the public health issues
4. Analyzing sentiments of news articles related to the public health issues
5. Learning topics (focuses) of news articles associated with the public health issues using topic modeling

**Figure 1** shows a schematic view of the main steps for mining Reuters news articles. Additional details about the main steps are discussed in the following sections. The Python scripts for mining news articles can be accessed via Github [1].

1. **Identifying Major Public Health Issues**

Major public health issues are generally studied and published in public health journals. Abstracts and keywords of the published articles convey their main research focuses. Thus, we developed the following procedure to identify major public health issues.

1. We selected 30 top journals on public health, which are listed in **Supplementary document 2**, together with their ranks, total citation numbers, impact factors, and eigenfactor scores [2].
2. We downloaded all the abstracts as well as keywords of articles published in these journals between January 1, 2007 and December 31, 2017 from the PubMed website by searching the journal titles in a given year [3]. We collected total 61,387 abstracts in the MEDLINE format.
3. We then matched these keywords to Medical Subject Headings (MeSH) terms [4]. MeSH is a controlled terminology developed by National Library of Medicine for indexing articles in the MEDLINE database. We then identified the synonyms of the matched MeSH terms using Unified Medical Language System (UMLS) Metathesaurus downloaded from the UMLS official website [5]. The UMLS Metathesaurus is a collection of many controlled terminologies and provides mapping structures between different medical vocabularies via concept unique identifier (CUI). Specifically, UMLS Metathesaurus assigns an identical CUI to a MeSH term and its synonyms. We looked up the CUI-MeSH mapping table in the UMLS Metathesaurus to map a MeSH term to its CUI and used the CUI to identify its synonyms.
4. We developed a Python script with regression expression [1] to identify the MeSH terms (and their synonyms) in the abstracts and calculated the frequencies of the MeSH terms. The frequency of a MeSH synonym is added to the frequency of the MeSH term.
5. After removing the MeSH terms which are not related to public health or whose relative frequencies are less than 1%, we found 90 popular MeSH terms on public health (See **Supplementary document 3**). We selected 10 major public health issues out of the 90 MeSH terms in terms of their frequencies (See **Table 1**).
6. **Collecting, Cleaning, and Filtering News Articles**

Reuters news agency is a leading global information news agency and the world’s largest international text and television news provider. We collected all the news articles between January 1, 2007 and December 31, 2017 from Reuters news agency for investigating major public health issues. We further cleaned these news articles, since these news articles contain noise and metadata that could affect the results of sentiment analysis and topic modeling toward public health issues. After that, we filtered news articles related to the 10 public health issues for sequential analysis. Details on collecting, cleaning, and filtering news articles are described in the following steps.

1. ***Collecting News Articles*.** We developed a web crawler in Python [1] and downloaded total 3,763,737 news articles from an online archive of Reuters news agency in the study period time [6].
2. ***Cleaning News Articles*.** After reviewing a small sample of news articles, we identified metadata patterns and noise that needed to be removed from the news articles. We deleted readers’ comments in the news articles because we focused on the content analysis of news articles. We removed the tags, such as “(Reuters)”, and editorial information, such as sentences started with “reporting by”, or “editing by”, using regular expressions. Similarly, we removed repetition of special characters, such as “---”, from the news articles. We replaced the sentence delimiters “*” and “>” using a period and replaced the hyperlinks, such as “http://topnews.session.com” with the word “link”.
3. ***Filtering News Articles.*** To filter news articles related to the 10 public health issues, we imported these news articles into Apache Solr [7] using Python API [8] for information indexing and searching. Apache Solr is an open source search platform with rich features for handling document, such as full-text search and real-time indexing. The Reuters news data was then filtered in Apache Solr to retrieve news articles that mentioned the 10 public health issues.
4. **Calculating Coverage of News Articles**

We performed descriptive statistics to calculate the coverage of news media (i.e., numbers of news articles) for the 10 public health issues over time. We then compared the coverage trends of news articles to those of Google Trends searches for the public health issues. We downloaded the Internet search pattern data of the individuals for the 10 public health issues from the website of Google Trends [9].

1. **Analyzing Sentiments of News Articles (Sentiment Analysis)**

We used a Python module, VADER [10] to quantify the sentiments of news articles toward the 10 public healthcare issues. To improve the accuracy of sentiment analysis for each public health issue, we only measured the sentiments of sentences containing MeSH terms and their synonyms. The specific steps for analyzing sentiments of news articles are:

1. We tokenized each news article into sentences using Natural Language Toolkit [11] and filtered sentences that mention the MeSH terms and their synonyms.
2. We calculated the sentiment score of each filtered sentence using VADER [10].
3. We calculated the average of sentiment scores of all sentences related to the public health issue in a news article as a sentiment score of the news article for the public health issue.
4. We computed the average sentiment scores of all sentences linked to a public health issue in all the news articles as a sentiment score of news media for the public health issue in each year.
5. We classified a news article as either positive, neutral or negative, according to the threshold values suggested by VADER. More specifically, if a sentiment score of a news article is equal to or larger than 0.05, the sentiment of the news article is positive; if a sentiment score of a news article is less than 0.05 and larger than -0.05, the news article has a neutral sentiment; otherwise, the news article is negative.
6. **Learning Topics of News Articles** (**Topic Modeling)**

Due to the limitations of traditional Latent Dirichlet Allocation (LDA) method (e.g., requesting a topic number as an input and ignoring important word order), we used an advanced topic modeling method, Topic Keyword Model (TKM) [12] to identify the hidden topic structure of news articles related to each of the 10 public health issues. We developed the following steps to perform topic modeling on news articles:

1. For each of the 10 public health issues, we retrieved news articles mentioning the public health issue in terms of MeSH terms and their synonyms for topic modeling.
2. We converted the words in the news articles into lowercases and stemmed and lemmatized these words with the NLTK [11] to reduce these words into their base forms.
3. We imported the processed news articles into the TKM package [12] to learn the topics of the news articles linked to each of the 10 public health issues. TKM will determine an appropriate number of topic number and output the learned topics of these news articles after reaching convergence criterion.

**References:**

1. Ming Huang, M.Z., Somaieh Goudarzvand, Sagheb Hossein Pour Elham, and Lixia Yao. *News Mining Source Codes*. 2019; Available from: <https://github.com/huangming6220/news_mining>.

2. Web of Science. *Journal Citation Reports*. 2019; Available from: <https://clarivate.com/webofsciencegroup/solutions/journal-citation-reports/>.

3. National Center for Biotechnology Information. *PubMed Advanced Search*. 2019; Available from: <https://www.ncbi.nlm.nih.gov/pubmed/advanced>.

4. National Library of Medicine. *Medical Subject Headings*. 2019; Available from: <https://www.nlm.nih.gov/mesh/meshhome.html>.

5. National Library of Medicine. *Unified Medical Language System (UMLS)*. 2019; Available from: <https://www.nlm.nih.gov/research/umls/index.html>.

6. Reuters. *Reuters news archieve*. 2019; Available from: <https://www.reuters.com/news/archive>.

7. Apache Software Foundation. *Apache Solr*. 2017 October 5 [cited 2017 October 5]; 7.0.1:[Available from: <http://lucene.apache.org/solr/>.

8. Didier Deshommes. *Solrcloudpy*. 2019; Available from: <http://solrcloudpy.github.io/solrcloudpy/>.

9. Google, I. *Google Trends searches*. 2019; Available from: <https://trends.google.com/trends/?geo=US>.

10. Hutto, c.j. *VADER Sentiment Analysis*. 2019; Available from: <https://github.com/cjhutto/vaderSentiment>.

11. NLTK Project. *Natural Language Toolkit*. 2019; Available from: <https://www.nltk.org/>.

12. Johannes Schneider, M.V. *TKM Package*. 2018; Available from: <https://github.com/JohnTailor/tkm>.

1. equal contribution [↑](#footnote-ref-1)
